# Minimally invasive surgery of the anterior skull base: transorbital approaches

**DOI:** 10.3205/cto000118

**Published:** 2016-07-11

**Authors:** Holger G. Gassner, Franziska Schwan, Karl-Michael Schebesch

**Affiliations:** 1 Department of Otolaryngology, Head & Neck Surgery, University Medicine of Regensburg, Germany; 2 Department of Neurosurgery, University Medicine of Regensburg, Germany

**Keywords:** anterior skull base, transorbital, endoscopic, approach, minimally invasive, tones

## Abstract

Minimally invasive approaches are becoming increasingly popular to access the anterior skull base. With interdisciplinary cooperation, in particular endonasal endoscopic approaches have seen an impressive expansion of indications over the past decades. The more recently described transorbital approaches represent minimally invasive alternatives with a differing spectrum of access corridors. The purpose of the present paper is to discuss transorbital approaches to the anterior skull base in the light of the current literature. The transorbital approaches allow excellent exposure of areas that are difficult to reach like the anterior and posterior wall of the frontal sinus; working angles may be more favorable and the paranasal sinus system can be preserved while exposing the skull base. Because of their minimal morbidity and the cosmetically excellent results, the transorbital approaches represent an important addition to established endonasal endoscopic and open approaches to the anterior skull base. Their execution requires an interdisciplinary team approach.

## 1 Introduction

For the past decades, a clear tendency to minimally invasive approaches has been observed in surgery of the anterior skull base. Technical development and interdisciplinary cooperation have made it possible to treat even complex pathologies. In this context, for example improvements of the video-endoscopic displays, the development of special instruments, improvement of imaging, and performing surgeries together in the 4-hands technique have become important.

Articles published by teams around Moe et al. [1] and Boahene et al. [2] contributed to establish a systematic of the method and to extend its indications. In principle, the incisions applied have been established for a long time, but their application in the presented concept is novel.

The aim of the present paper was to present the relatively new technique of the transorbital endoscopic approaches and to evaluate them based on the current literature. The article focuses on the following aspects:

- Soft tissue and skeletal anatomy of the transorbital approaches to the anterior skull base
- Ability and limitations of exposing the anterior skull base
- Risk and complication profile of those approaches from an ENT-specific and neurosurgical point of view
- Indications of those approaches from an ENT- and neurosurgical perspective

Aspects that have no particular relation to the transorbital approach but only have a general relation to skull base surgery, are mentioned only with regard to topic-related aspects. The character of the present article is different in that its focus is placed on the introduction of a relatively new method. Compared to long established methods the literature on this topic is rather compact. The relevance of the topic results from the therapeutic options that for frequent pathologies in the field of otolaryngology, including surgery of the anterior and posterior wall of the frontal sinus.

## 2 Anatomy

### 2.1 Eye lids

The medial canthal ligament consists of a superficial anterior and a deeper posterior part. The superficial anterior part originates from the superficial muscle heads of the pretarsal and preseptal parts of the orbicularis oculi muscle. It inserts at the anterior aspect of the lacrimal crest. The deep posterior part of the medial canthal ligament originates from the deeper parts of the orbicularis oculi muscle and inserts at the posterior aspect of the lacrimal crest. Detachment of the anterior part is generally compensated, if the insertion of the posterior part remains intact. The lateral canthal ligament (the authors will not discuss the controversial question if it is a ligament, a tendon, or a muscular condensation) originates at the lateral edge of the tarsi from fibrous and muscular condensations. The precanthal ligament is a thickened part of the orbital septum and inserts at the periosteum of the ascending branch of the zygomatic arch. Following lateral canthotomy, this precanthal ligament, and further periosteal insertions have to be transected when performing inferior cantholysis in cases of acute treatment of orbital compartment syndrome. The muscular part originates from the pretarsal parts of the ocular orbicularis muscle and inserts as tendon at Whitnall’s tubercle [3], [4], [5], [6], [7], [8], [9], [10], [11], [12].

### 2.2 Bony anatomy

#### 2.2.1 Infrabasal anatomy

The orbit is a bony cavity which open to the front. Its bony edges consist of the frontal bone, the lacrimal bone, the maxilla, the zygomatic bone, the ethmoid bone, palatine bone, and the sphenoid bone. The roof of the orbit and thus the anterior skull base is formed by the frontal bone and to a small portion in the posterior area the small wing of the sphenoid. The trochlear fovea contains the trochlea which is highly significant for the transorbital approach to the skull base. The lacrimal gland adheres to the lacrimal fossa. The eyelids represent the limitation of the eye cavity in frontal direction.

#### 2.2.2 Suprabasal anatomy

The cranial skull base is similar to a plane surface that is divided by the rhinobase with the ventrally located crista galli where the cerebral falx and the superior sagittal sinus inserts intradurally [13], [14], [15]. Since the drainage of the bridging veins merging into the superior sagittal sinus is not very high [16], [17], the sinus can be ligated in its anterior third without any problem, it can be transected, and removed in order to expose the posterior surface of the frontal sinus and the transition to the frontal skull base [18]. In lateral direction, the frontal skull base merges into the calvaria of the convexity, in rostral direction the concave small wing of the sphenoid bone delineates the posterior limit of the anterior skull base together with the anterior clinoid process [15]. Medial to the anterior clinoid process, symmetrically on both sides the optic canal with the optic nerve is found accompanied by the ophthalmic artery. The intracanalicular course of the optic nerve measures about 10 mm. The anterior part of the sella turcica finally merges into the planum sphenoidale which marks the medial and rostral point of the frontal skull base.

At the level of the lamina cribrosa, the thin olfactory fibers transdurally, form the olfactory nerve and bulb that extends nearly along the whole length of the frontal skull base to the limbic system.

The frontal lobe is lying on the frontal skull base. At the level of the olfactory grooves, the straight gyrus is found. The brain is completely covered by dura, which strongly adheres to the bone. The dural and bony blood supply originates mainly from branches of the ethmoid arteries, i.e. from terminal branches of the external carotid artery. The brain itself, including adnexal structures (e.g. cerebral nerves and pituitary gland [19]), receives its blood supply predominantly from branches of the anterior and medial cerebral artery, i.e. from the internal carotid artery [15].

## 3 Definition and objective

The term transorbital implies access through the orbit, i.e. an approach entering and leaving the orbit in order to reach the surgical target. Generally such an approach can be performed via numerous incisions that have all been established for a long time. Already in 1998, Harris et al., for example, described their 12-year experience of lateral orbitotomy performed by upper eyelid incision in 600 cases [20], [21], [22] [23].

The articles published by the team of Moe et al. and Boahene et al. contributed significantly to establishing a systematic concept of the minimally invasive approaches. Since the method has been developed based on long-established techniques and incisions and further important publications have been written parallel, a first description cannot be exactly defined and this aspect is also not the purpose of the current paper [1], [2].

### 3.1 Approaches: systematics

**Figure 1:**
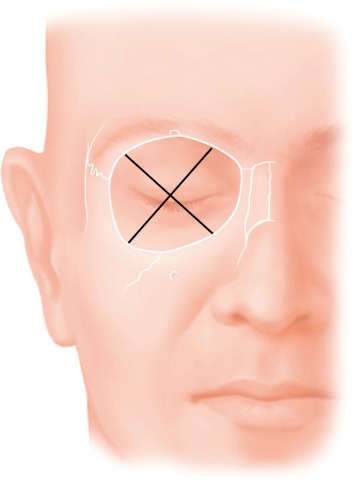
Division of the orbit into 4 quadrants. The superior lid crease approach allows access to the superior quadrant, the precaruncular incision allows access to the medial quadrant, the lateral retrocanthal incision allows access to the lateral quadrant, and the transconjunctival incision allows access to the inferior quadrant.

Moe et al. describe approaches to the 4 quadrants of the orbita under the term of “TONES”, which stands for “transorbital neuroendoscopic surgery”. Since Moe’s classification of incisions is most appropriate to analyze transorbital approaches in a structured way, this classification will be taken as basis for this manuscript [1], [2]. As presented in Figure 1 (Fig. 1), the approaches are divided according to the quadrants.

1. Approach to the medial quadrant: precaruncular approach
2. Approach to the superior quadrant: upper eyelid approach
3. Approach to the lateral quadrant: lateral retrocanthal approach
4. Approach to the inferior quadrant: transconjunctival approach

The location of the pathology represents an important criterion for the choice of the best approach. Moe et al. distinguish between the interorbital and the supraorbital corridor which is demarked by the coronal tangent through the medial equator of the bony orbit. Surgical targets in the supraorbital corridor are often treated via the superior lid crease approach, targets in the interorbital corridor are treated via the precaruncular approach [1].

### 3.2 Defining the topic

The objective of the current manuscript is to analyze transorbital approaches that are characterized by particularly esthetic incisions, minimal soft tissue dissection, and reduced manipulation of the bone. Procedures that are associated with visible scars are excluded from the present discussion. Incisions are limited to transconjunctival and upper eyelid incisions. The lateral extension of the upper eyelid incision along a crow’s foot up to the orbital rim is also included, since the resulting scar remains generally invisible. Extensions as described by Abdel Aziz, 2.5 cm lateral of the lateral canthus, extending beyond the bony rim of the orbita are excluded [24]. Furthermore, transcutaneous lateral canthotomy and trans- and parapalpebral incisions are also excluded.

Inclusion criteria are:

1. The incision is limited to the conjunctiva, the skin of the upper eyelid, and a skin fold over the lateral orbital rim.
2. Craniotomy is performed through the thin bone of the orbital roof and not through the calvarial bone.
3. The use of endoscopic optics contributes to the minimally invasive character of these approaches. Important structures can be illuminated via small accessory corridors, they are visualized, and can be manipulated with special instruments. Also video endoscopy contributes significantly to the surgical training [20].

### 3.3 Approaches: surgical techniques

#### 3.3.1 The precaruncular approach

**Figure 2:**
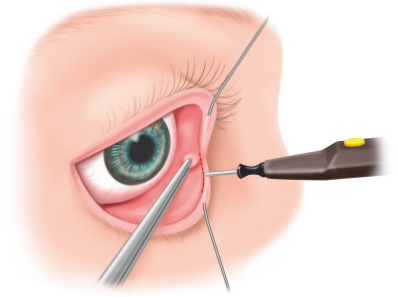
The precaruncular incision is performed after intubation of the lacrimal canaliculi and retraction with lacrimal duct probes; the caruncula is retracted in medial direction with forceps, the precaruncular incision is performed with a fine monopolar needle.

The precaruncular approach opens the medial quadrant and allows access to the lamina papyracea, the anterior and posterior ethmoid arteries, and the interorbital corridor of the anterior skull base. The technique described by Moe creates an access posterior and lateral to the lacrimal ducts (Figure 2 (Fig. 2)). After inserting a bulb protector, the lacrimal duct probes are inserted in both canaliculi and allow retraction and protection of those structures. The globe is carefully moved in lateral direction by means of a spatula, the caruncula is retracted in lateral direction with small forceps and released with a fine monopolar needle medial along the skin-caruncula border. The incision is extended into the conjunctiva of the upper and lower eyelids and the medial canthal ligament is exposed. The technique described by Raza and Boahene draws the incision in further cranial direction.

**Figure 3:**
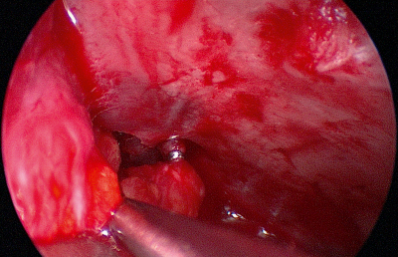
Precaruncular exposure of the anterior ethmoid artery. The artery is clipped, the fracture line inserts from posterior into the anterior ethmoid foramen.

**Figure 4:**
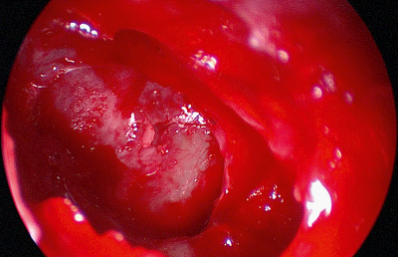
Exposure of the dural defect after defragmentation of the fracture line.

Posterior of the insertion of the medial canthal ligament at the crista lacrimalis, the periorbita of the lamina papyracea can now be incisied. The height of the roof of the rhinobase is revealed by the anterior and posterior ethmoid arteries. After clipping the ethmoid arteries, endoscopically controlled resection or clearing out of the medial orbital roof allows exposure of the anterior skull base (Figure 3 (Fig. 3), Figure 4 (Fig. 4)).

#### 3.3.2 The superior lid crease approach

**Figure 5:**
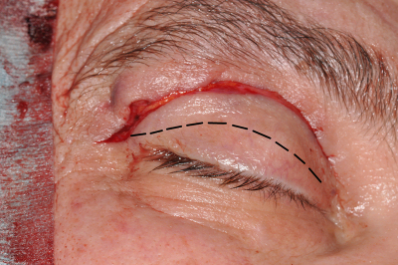
“Classical” supratarsal incision (dashed black line); the authors prefer the supra-supratarsal incision (actual incision line) which allows enhanced exposure of the anterior skull base with excellent and generally invisible scarring.

The superior lid crease approach is also called upper eyelid approach. Moe et al. and Boahene et al. describe the skin incision in the supratarsal fold. The authors of the present paper favor an incision about 7–12 mm above the supratarsal fold. This “supra-supratarsal incision” remains located in the thin skin of the upper eyelid and avoids the skin of the eyebrow that is clearly different with regard to texture and color. The higher supra-supratarsal incision corresponds to the superior incision of a cosmetic blepharoplasty, just like the supratarsal incision it generally remains without visible scars (Figure 5 (Fig. 5)). The access corridor to the anterior skull base becomes larger due to the more superior placement of the incision, a lateral extension to the crow’s feet is still possible. The mainly sub-periosteal access to the orbital roof is medially limited by the trochlea and laterally be the canthal ligament [1], [2].

With technically correct execution, subperiosteal release of the trochlea is safely possible feasible and considerably extends the approach to the anterior skull base, especially after clipping the ethmoid arteries. Reconstruction of the trochlea is performed by simple repositioning, suture fixation is not necessary. This is reported by Haug et al. in a retrospective study performed on 15 patients who underwent reconstruction of the subperiostally removed trochlea by reposition of the orbital soft tissues. The function of the extraocular muscles was intact in all patients [25]. The authors of the present manuscript made the same experience in 12 patients who underwent removal of the trochlea for supraorbital access to the anterior skull base. Raza et al. reported one patient who had diplopia after removal of the trochlea that turned out to be self-limiting. As a consequence of this observation, those authors have subsequently avoided the subperiostal removal of the trochlea [2].

**Figure 6:**
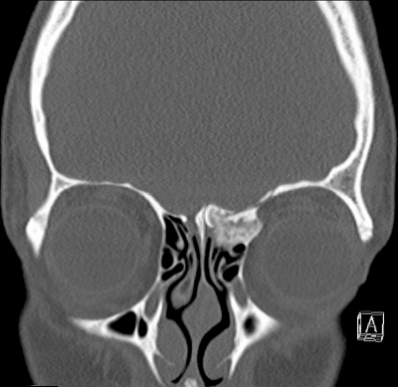
15-year-old female patient with symptomatic recurrence of a monostotic fibrous dysplasia status post transcranial removal 3 years previously. The patient presented with increasing diplopia and cephalgia.

**Figure 7:**
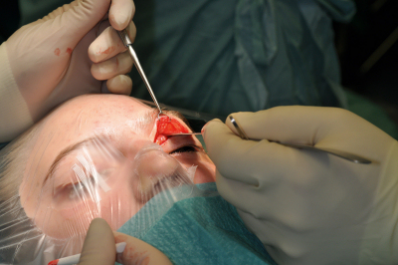
Transorbital upper eyelid approach. The pathology was completely resected after release of the trochlea and clipping the anterior ethmoid artery.

**Figure 8:**
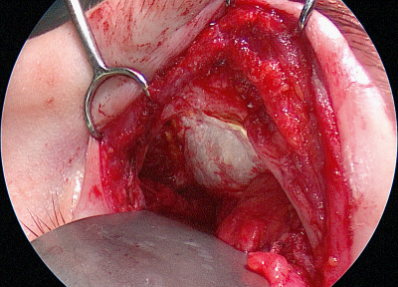
The resulting dura defect was covered with Tachosil^®^.

**Figure 9:**
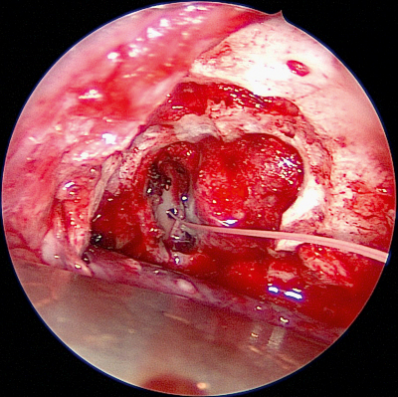
Because CSF leak became clinically evident, the patient had to undergo revision surgery on the second postoperative day. Fascia lata and abdominal fat were placed into the defect with bone anchors in the Ccista galli and the lateral orbital roof.

**Figure 10:**
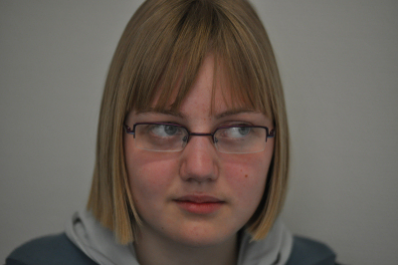
After 2 years follow-up, the patient is recurrence-free. The diplopia resolved almost completely and does not affect her daily life. The cephalgia persisted and is controlled with single agent medical therapy.

Figure 6 (Fig. 6), Figure 7 (Fig. 7), Figure 8 (Fig. 8), Figure 9 (Fig. 9) and Figure 10 (Fig. 10) illustrate the potential of the upper eyelid approach with removal of the trochlea. The recurrence shown in Figure 6 (Fig. 6) of a monostotic fibrous dysplasia that was transcranially resected 2 years previously manifested by increasing diplopia and displacement of the bulb. The lesion encompassed the bony base of the trochlea and the trochlear fossa. As depicted in Figure 7 (Fig. 7), the trochlea was released through the superior eyelid approach and the lesion was resected. After duraplasty (Figure 9 (Fig. 9)), persistent CSF rhinorrhea was observed and revision via the same access became necessary on the first postoperative day. Bone anchores were placed in the crista galli and the lateral orbital roof. With these sutures, a duraplasty of abdominal fat and fascia lata was secured (Figure 6 (Fig. 6), Figure 7 (Fig. 7), Figure 8 (Fig. 8), Figure 9 (Fig. 9), Figure 10 (Fig. 10)).

Lateral extension: The lateral orbital wall and apex can be exposed by detaching the lateral canthal ligament. A detachment of the lateral canthal ligament is performed in the subperiostal layer and does not require lateral canthotomy. Reconstruction is performed with a canthoplasty as described by Moe. The lateral canthal ligament is looped with sutures and fixed via drill holes perforating the zygomatico-frontal suture [1].

**Figure 11:**
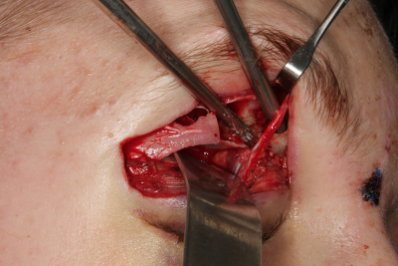
Access to the posterior wall of the frontal sinus and further to the cribriform plate and the crista galli after temporary removal of the anterior wall and the floor of the frontal sinus. The supraorbital nerve is preserved and protected.

**Figure 12:**
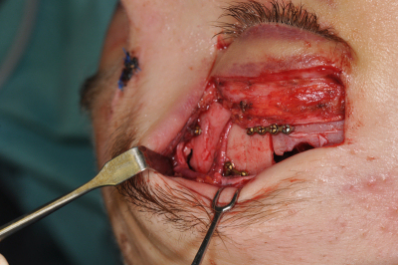
Reconstruction of the continuity of the anterior wall and the floor of the frontal sinus by micro-plate osteosynthesis.

**Figure 13:**
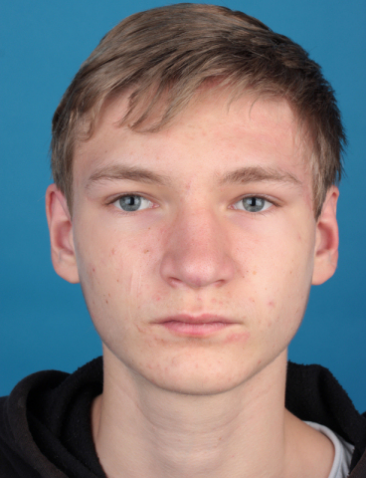
16-year-old patient one year after transorbital management of an anterior skull base fracture with defect covering of the right cribrosa plate via an upper eyelid approach. Bone contour (Figure 12), function of the extraocular muscles, sensitivity, and cosmetic appearance are without deficit.

Superior extension: The temporary removal of the anterior wall and floor of the frontal sinus allows access to the posterior wall. The anterior wall segment can be split sagittally at the level of the supraorbital foramen in order to protect the supraorbital nerve (Figure 11 (Fig. 11)). Access to the posterior aspects of the interorbital corridor is created and to the lamina cribrosa and the crista galli are exposed as described in the presented case. Resection of the frontal sinus septum in the sense of Draf III surgery can also be performed in this way. The anatomical continuity of the frontal sinus walls is restored by osteosynthesis with micro-plates (Figure 12 (Fig. 12)). Figure 13 (Fig. 13) illustrates the postoperative result of the 16-year-old patient one year after dural repair with unimpaired function and cosmetic appearance.

#### 3.3.3 The lateral retrocanthal approach

**Figure 14:**
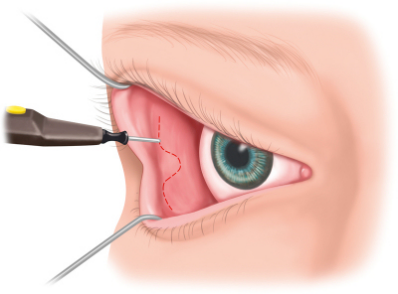
The retrocanthal incision preserves the insertion of the lateral canthal ligament at Whitnall’s tubercle. The incision is performed as lateral extension of the transconjunctival lower eyelid incision, curving superiorly behind the lateral canthal ligament.

The lateral retrocanthal incision is performed in the conjunctiva posterior to the insertion of the lateral canthal ligament at Whitnall’s tubercle (Figure 14 (Fig. 14)). It allows for example osteosynthesis of a fracture of the zygomatico-frontal suture without external skin incision or lateral decompression of the orbit [26]. Comparative clinical studies of this type of incision have not be published up to now. Bly et al. showed in a combined anatomical and computer-simulated study that this approach allows accessing further centrally located areas such as the lateral cavernous sinus and the middle cranial fossa [27].

#### 3.3.4 The transconjunctival approach

The transconjunctival approach to the orbital floor is preferred by many authors including the authors of this manuscript and considered as excellent access for example for treatment of fractures of the orbital floor [22]. Its use for access to the skull base is limited. 

#### 3.3.5 Transorbital craniotomy and craniectomy

The term craniotomy describes a procedure where a bone cover lying on the dura is temporarily removed and re-inserted after intervention. Craniectomy is a procedure where the bone cover lying on the dura is permanently removed. The access to the dura through the orbital roof is generally performed as craniectomy, in studies published up to now the resected bone is not re-inserted. A possible consequence is the development of a pulsatile exophthalmos. In available studies, the pulsatile exophthalmos was described as self-limiting.

Andaluz et al. and Abdel Aziz et al. describe the resection of a superolateral orbital segment via an upper eyelid approach which is performed in a curved line lateral to the lateral canthus over the orbital rim. The segment incorporates the orbital rim and the fronto-zygomatic suture and extends to the fronto-sphenoid suture. Andaluz et al. treated 5 aneurysms of the anterior circulation and 3 tumors of the anterior skull base via this access. Abdel Aziz et al. treated 40 patients, among those 31 patients suffering from aneurysms. The resection of a segment of the anterior wall of the frontal sinus has been described as well. Complications were not observed, the cosmetic results were excellent. After repositioning, the bone segments were fixed by plate osteosynthesis. A systematic regarding the size and position with corresponding nomenclature of the bone segment to be removed has not been described until now. Most authors report that the bony access is performed in an individualized way, depending on the pathology [24], [28].

## 4 Indications and limitations

### 4.1 Preliminary remarks: open vs. transorbital approaches – from a neurosurgical perspective

The indication for an open neurosurgical (microsurgical) approach depends on the expertise and the interdisciplinary cooperation of ENT and neurosurgery [29], [30], [31], on the comorbidities and the age of the patient [32] as well as the individual patho-anatomy [13], [33], [34]. In their review article, Marchal et al. described variations of access depending on the anatomy of the lesion in an interdisciplinary setting [29] and Zimmer et al. described different technical and equipment-related options of minimally invasive or endoscopic as well as open transcranial accesses to the lesions of the anterior skull base [31].

Hendryk et al. investigated the postoperative outcome of 15 patients with neoplasms of the anterior skull base that were operated either transcranially or transfacially. In this population, a better functional outcome was observed after transcranial surgery of benign lesions where additional reconstruction of the dura and the bony base was necessary, whereas malignant lesions could be better reached en bloc via a transfacial access and had a better functional outcome [33].

In summary, however, all authors emphasize that a dogmatic approach to complex lesions of the anterior skull base is not appropriate. This statement is important in the context of different surgical expertise, progresses in technology, and developing treatment concepts [29], [30], [31], [33], [34] that question the radicalism still postulated some years ago at the prize of functionality [35].

The authors Zimmer et al., Rawal et al. and Husain et al. emphasize in their current papers that generally a significantly less traumatizing transorbital or transfacial approach should be preferred for resection of a tumor, repositioning of a fracture, or covering of a fistula when possible [31], [36], [37], [38].

The team around Kris Moe from Seattle, USA, published 3 important papers from 2010–2012 describing the transorbital approach to the skull base and characterizing the surgical-technical procedures [1], [39], [40]. The undisputed advantage of this approach is clearly its minimally invasive character and at the same time the possibility to visualize large parts of the anterior skull base and to be able to manipulate them (endoscopically assisted, if necessary).

Since the TONES approach [1] could not yet be investigated in a controlled way on a larger scale, only data about the classical cranio-facial access or its neuro-surgical equivalent exist, i.e. the subfrontal (bi-/unifrontal) access. As mentioned above, it depends on the surgical expertise of the interdisciplinary team which surgical procedure is preferred.

For certain pathologies, it is certainly wise to be prepared to switch from a minimally invasive to an open approach, either for exposure or for urgent intervention. Being prepared in all regards is especially advisable when bone or sometimes dura infiltrating carcinomas [33], [38], sarcomas [41] or metastases [42], para-ophthalmic carotid aneurysms [43], but also in meningiomas growing through the skull base [33], [44], or rare tumors such as for example esthesio-neuroblastoma are present [34], [45]. Also in cases of empyema, abscesses, fractures, and dural lesions it might be required to change from a transorbital access to a combined neurosurgical approach [13], [31].

An operative algorithm is currently being evaluated by the authors.

### 4.2 Comorbidities and other patient-specific factors

As it is necessary with every surgical intervention, the individual risk profile of the patient with all relevant comorbidities has to be evaluated, especially with regard to postoperative pain management [46] and, as described by Salmaggi et al. and Rolston et al. in their current publications, to perioperative thrombo-embolic events [47], [48]. 

Regarding the surgical planning of the approach and the parenchymal rigidity, it is particularly relevant to assess the status of previous surgeries in the area of the skull base and the frontal sinus [49], [50]. Generally, each previously performed surgery in this area bears an increased risk of perioperative morbidity due to scarring and adhesions in the area of the basal dura and the dura-adjacent cortex. In a current analysis performed by Harvey et al. in 106 patients having undergone endoscopic interventions of the skull base, the factor of “revision surgery” was identified as being a significant predictor of surgical complication (p=0.003) [50]. Those patients should always be informed specifically – for medico-legal purposes [51].

When manipulations at the frontal brain are expected, for example with intradural infiltration of the skull base, a perioperative anti-osmotic medication (e.g. steroids, mannitol, hyperosmolar saline) is recommended to reduce swelling of the brain after mechanical irritation [52], [53].

This fact is also taken into consideration in the current guidelines of the neurooncological societies and finally published as EANO guideline for high-grade gliomatous tumors in Lancet Oncol [54].

Furthermore, generally a perioperative antibiotic prophylaxis (e.g. cephalosporins) is recommended [55], [56], [57] that should be repeated after 8 hours of surgery [58].

Perioperative anti-convulsive therapy (e.g. with levetiracetam [59], [60]) is controversially discussed. However, especially in cases of temporal and frontal manipulation – in the context of transcranial interventions – this measure should be considered because those cerebral areas are considered as being particularly ictogenetic and vulnerable for manipulations [58], [60].

In summary, however, with reference to a transorbital approach without direct manipulation of the cerebral parenchyma, no general recommendation for anti-convulsive prophylaxis is expressed because no data is available justifying the administration of accompanying anti-convulsive medication.

## 5 Diagnostics

### 5.1 Imaging

The basis for planning surgery and approach is the radiological imaging. It has the highest significance for the decision if a transorbital, endonasal, transfacial, and/or transcranial approach is chosen.

Patients after cranio-cerebral trauma with indication for surgical revision of the skull base should undergo thin-layer computed tomography. In order to exactly plan and perhaps even simulate the procedure, it is recommended to display the primary dataset in a coronal as well as sagittal reconstruction [61]. Especially with regard to the TONES approach, the target and exposition surface should be displayed in all three levels, also because the most direct pathway (precaruncular, preseptal, retrocanthal) should be chosen in order to minimize the orbital compression as much as possible [1].

MR imaging, generally with contrast enhancement, should always be performed in oncological and inflammatory processes [62], [63] as reference for the extent of the resection and for follow-up. Native preoperative thin-layer CT scan is completed in order to reveal the bony structures with regard to a possible infiltration or penetration of the skull base and/or ethmoid cells and paranasal sinuses [61].

### 5.2 Histological diagnosis

Histological diagnosis is an essential precondition for planning of surgery and possible adjuvant therapy. Furthermore, in case of infiltrative processes the resection should be performed with clear margins on frozen section control. Of course, this is recommended for both the transorbital and the transcranial approach.

In cases of intradural soft tissue tumors larger samples of tissue are taken from the vital tumor margins because in addition to classical histological diagnosis and WHO grading, modern pathological examination identifies and quantifies important molecular markers (e.g. Ki67 labeling index, Ip19q, LOH) for adjuvant therapy [64], [65] in order to allow individualized neurooncological multimodal therapy [65].

## 6 Preoperative management

### 6.1 Interdisciplinary planning

Increasingly, interdisciplinary cooperation becomes the standard in the management of pathologies that require complex diagnostic and multimodal treatment strategies. The treatment of malignant tumor diseases is more and more planned and accompanied by interdisciplinary tumor boards. Also for skull base pathology, interdisciplinary approaches are gaining in importance. Data on the effectiveness of interdisciplinary cooperation in the management of skull base pathology are scarce. Lutterbach et al. analyzed 1516 patients who had been discussed over 15 years at Freiburg, Germany, in the interdisciplinary brain tumor board. About one third of those cases were affected with neoplasms of the skull base. 91% of the therapeutic recommendations were implemented in this patient population. The authors draw the conclusion that an interdisciplinary treatment recommendation is implemented very reliably [66]. McLaughlin et al. analyzed in a review article the advantages of interdisciplinary cooperation and draw conclusions on the management of diseases of the skull base. Those authors argue that an interdisciplinary cooperation has important advantages for all aspects of the treatment of patients with skull base diseases, including the clinical diagnosis, imaging, invasive diagnostics, and especially for the selection and performance of the necessary therapeutic modalities. The authors recommend a structured and formalized process for establishing and managing an interdisciplinary team [67]. Specific data on the management of minimally invasive approaches are not available up to now.

## 7 Perioperative management

### 7.1 Positioning

Regarding the positioning of the patient, the general question must be asked if fixation of the head in a Mayfield clamp is indicated. In a study published by Andaluz et al. 5/5 patients were fixated [28]. In this study, neither negative nor positive consequences of this measure are described.

The advantages include the well-known risk profile of this measure (bleeding, infection, scarring, alopecia). Furthermore, the access can sometimes be difficult for the instruments, and it is difficult for the surgery team to work in an ergonomic posture. The key advantage is the ability to switch to an open transcranial approach more quickly. Especially in the context of pathologies that require a swift change, as for example the clipping of aneurysms of the anterior circulation, fixation of the patient’s head is certainly recommended.

### 7.2 Neuronavigation

For intraoperative localization of the tumor, or the fracture, neuronavigation is a helpful tool [68], [69], that may allow to reach the surgical target in the shortest and least traumatizing way [70].

For non-parenchymatous extra-axial or bone tumors of the frontal or temporal skull base, navigation remains precise during the entire procedure. According to a recent publication, 235 endoscopically experienced neurosurgeons routinely apply this tool for skull base surgery [71]. Even for lesions of the clivus and the paranasal sinuses that can be accessed by a craniofacial approach, neuronavigation was reliably applied [72]. In a cadaver study, Feigl et al. showed that neuronavigataion is also precise in keyhole approaches to the anterior skull base [73]. No data prospective data analyzing its effectiveness for the TONES approaches herein have been published to date. 

With resection of cerebral soft tissue lesions, neuronavigation faces important limitations. A brain shift occurs after craniotomy, release of liquor, and consecutive change of all intracranial pressure [74]. Its value for planning is maintaines even in these cases, because less movable surrounding structures like dura and bone still act as important reference (CT scan or MRI) [75], [76].

In the majority of cases, fixation of the skull in a Mayfield clamp is required for neuronavigation. Frameless systems may become more prevalent in the future [77], [78]. The reference system is usually fixated to the head clamp. This may make intraoperative handling difficult, especially when uni- or bifrontal approaches are planned. Advantages and disadvantages are weighed on an individualized basis [79].

### 7.3 Intraoperative imaging

Intraoperative MRI imaging with adapted field intensity (0.5–3.0 Tesla) is becoming increasingly important in surgical neurooncology [80]. Numerous randomized and prospective studies showed that the completeness of the resection and simultaneous preservation of the neuronal functionality could be significantlyenhanced [81]. For the anterior skull base, such specific data are not available at the current time [82], [83]. Intraoperative CT scan on the other hand has been shown to be beneficial in anterior skull base surgery, as this aides with precise identification of bony landmarks [84], [85], [86].

Of course, intraoperative CT scan appears also desirable for TONES. Also here prospective data are missing that evaluate the effectiveness and feasibility at this time.

Another procedure that is currently more and more in the focus of neurooncological neurosurgery, is fluorescence-assisted microsurgery [87], [88]. It is mainly applied for improved resection of brain tumors and cerebral metastases [89], [90] but it was also evaluated as being effective for dura-infiltrating tumors of the skull base [91]. Even in the context of the discussed transorbital approaches to tumors of the skull base, the fluorescence-assisted resection with Sodium fluorescein under an according light filter (YELLOW 560 nm, Carl ZEISS Meditec) may become an important adjunct.

### 7.4 Shift to transcranial approach

Intraoperatively it may become necessary that the endoscopically assisted minimally invasive transorbital approach must be changed to an open neurosurgical access via a uni- or bifrontal or even pterional (fronto-temporal) craniotomy [92], [93], [94], [95], [96]. Accordingly, the preoperative planning should always be performed in close cooperation with the involved disciplines, ENT and neurosurgery. OR and nursing staff should be included in the surgical plan [96], [97].

If the switch to an open transcranial neurosurgical procedure is possible, positioning of the patient, especially of the head should be performed in presence of a neurosurgeon. The fixation of the head in the Mayfield clamp should be completed prior to draping.

#### 7.4.1 Indications

The indication to change from a transorbital to an open neurosurgical procedure may be necessary because of inadequate exposure, rigidity or marked vascularization of the tumor [96], [98], in cases of larger dura defects, and in cases of intradural or parenchymatous (iatrogenic) hemorrhage [99]. If fractures of the rhinobase/orbitobase with consecutive relevant ruptures of the basal dura are significantly dislocated and complex, it may be necessary to cover the skull base from cranial with autologous or allogeneic material (see 7.4.2) [100], [101], [102], [103].

Generally it is mainly the expertise of the surgical team that determines how in the context of a TONES approach bleeding can be controlled and complex tumors and fractures of the skull base are considered accessible.

The study of the TONES approach published in 2010 by Moe et al. evaluates 20 TONES approaches performed in 16 patients to treat CSF fistulas,tumors, and fractures of the skull base without changing to an open neurosurgical procedure. However, the authors emphasize that neurosurgical craniotomy may be required at any time [1]. The authors of the present paper as well encountered no need to switch to a transcranial approach in 16 cases. In 1/16 cases, a two-stage transcranial procedure was necessary because the pathology compressing the optic nerve could not be reached satisfactorily.

#### 7.4.2 Procedure

##### Uni-/bifrontal (subfrontal) craniotomy

Via a bifrontal craniotomy, the entire anterior skull base can be visualized, via a unifrontal craniotomy the respective side is reached. These approach allow for excellent exposure; however, they are more traumatic and invasive. The pathology can be reached through an extradural or intradural pathway. Exposure allows access to the nasal cavity, the ethmoid complex, the rhinobase, and the medial, lateral, and superior orbital walls.

The merely extradural approach is mostly recommended in factures of the anterior skull base, complex fractures of the posterior wall of the frontal sinus, and tumors that do not infiltrate the basal dura [101].

The frontal lobe is better retracted through the intradural access. Consequently exposure and access for instruments are less limited. Retraction has to remain measured in order to minimize the risk of injury of the olfactory fibers at the level of the cribriform plate.

After slow retraction of the frontal lobe and release of CSF, the intradural route allows access to both optic canals, both optic nerves, the optic chiasm, the cranial pituitary stalk, both internal carotid arteries, the planum sphenoidale, and both anterior clinoid processes.

This approach requires exact anatomical orientation and surgical experience. The spectrum of complications is wide and extends from venous congestions to CSF rhinorrhea, and irreversible damage of the frontobasal cranial nerves.

##### Pterional (fronto-temporal) craniotomy

Classified as a variation of the subfrontal access, the pterional craniotomy is one of the standard open neurosurgical accesses. Nearly the entire ipsilateral and important parts of the contralateral skull base are exposed. After opening of the Sylvian fissure (fronto-temporal sulcus), the supra-, para-, and retrosellar region with all vessels and brain nerves including the optic chiasm, the optic tract, and the 3^rd^ ventricle are reached [104]. This approach is appropriate for pathologies in the area of the cavernous sinus, the lateral orbita, and especially for accessing the superior orbital fissure with its content (N. III, N. IV, N. V1, N. VI, ophthalmic vein).

##### Repair of skull base defects

As described above, complex fractures of the anterior skull base often require repair of the dural defect from cranially [105]. For this purpose, an appropriately sized galeal flap is preserved. This flap is pedicled caudally and receives its hemoperfusion mainly form the supraorbital and supratrochlear bundles. This flap can be dissected long enough to cover the planum sphenoidale, the roof of the sphenoid sinus and the superior ethmoid cells. The flap may also be used as a free graft. In every case, fixation at the edges of the dura with micro-sutures, fibrin glue, and/or material containing collagen fibers is recommended. If it is not possible to cover the defect with autologous material, allogeneic dura substitutes may be applied. However, many authors describe a higher risk of persistent csf leak [106].

##### Lumbar drainage

The placement of a lumbar drain for reduction of CSF pressure decreases the incidence of persistent postoperative CSF leakage. When a dural defect is anticipated to require relevant retraction of the frontal lobe, preoparative placement of a lumbar drain is recommended. This is facilitates intraoperative retraction of the frontal lobe [107], [108]. This is in contrast to older publication that stress the risk of insufficient suprabasal coverage of the CSF leak with insufficient CSF pressure on the sealing material. Additional risks are cited [108], including iatrogenic meningitis and encephalitis [109], [110], headaches associated with decreased CSF pressure pressure, intracranial hypotension [111], subdural hygromas [112] and hematomas [113], [114] as a consequence of a rupture of a dural bridging vein [115].

An absolute contraindication of lumbar drainage is a manifest stenosis at the level of the cerebral aqueduct, the 4^th^ ventricle, or in the foramen magnum. This may result in a lethal tentorial or foraminal herniation [108], [110]. Especially trauma patients require imaging with the question of patency of the CSF outflow tract [112].

## 8 Postoperative management

### 8.1 Intensive care surveillance

After craniotomy, generally ICU surveillance required is required for 48 hours. Ideally, the patients are extubated in the operating room so that the postoperative surveillance can be performed in the neurologically assessable patient. Clinical checks of neurological function of the cranial nerves, vigilance, and motor skills should be performed at regular intervals [97]. A focal neurological deficit that has not been documented preoperatively and that is not explained by the surgical procedure should always be worked up with immediate imaging, generally with a CT scan [116]. Complications during the early phase after craniotomy include intra- or extracerebral hemorrhage and contusions [117], CSF fistulas [118], and a tension pneumo-cephalus [119]. Relevant hemorrhage occurs with an incidence of 0.8–1.1% according to the neurosurgical literature [117], [120]. Depending on the mass effect, an emergent re-craniotomy with evacuation of the hematoma and, if needed, implantation of an intracerebral pressure probe and/or ventricular drainage may be indicated.

### 8.2 Clinical checks and imaging

Immediate postoperative care includes clinical checks of vital parameters, vigilance, cranial nerve function, motor innervation, amount and quality of drainage, wound healing, as well as postoperative laboratory control (CRP, blood count, electrolytes). If necessary, analgesia and nasogastric feeding are started [121], [122].

Type and frequency of postoperative is mainly determined by the biological activity of the tumor (WHO grading, histology, Ki-67 index), other suspect lesions (e.g. distant metastases), and the completeness of the resection. Generally CT scan and MRI – with contrast enhancement are obtained [116]. These may be complemented by nuclear medical (PET, SPECT) or specific sonographic variants [123].

In case of completely reesected benign skull base lesions, such as WHO class I meningiomas, a non – contrast CT scan is performed 48 hours postoperatively in order to exclude relevant postoperative hemorrhage. 12 weeks postoperatively, contrast enhanced MRI for documentation is obtained to document the status of resection. These are repeated in annual intervals for at least 5 years. In cases of malignant processes, contrast enhanced MRI is obtained even if complete resection can be supposed in order not to delay adjuvant therapy (e.g. radio-/chemotherapy) – depending on the residual tumor stage [123]. The follow – up MRI controls should then be performed in intervals of 3–6 months.

### 8.3 Rehabilitative measures

Generally, rehabilitation is required after resection of skull base pathology [124], [125]. The decision is individualized, taking into account factors including age, general health, and the need for adjuvant therapy. Adjuvant therapy is not delayed and rehabilitation is usually begun after completion of that therapy. 

The main aim of rehabilitation is reintegration of the patient into the daily routine and professional life. Losses (e.g. anosmia, diplopia, visual loss, frontal brain syndrome) should be compensated as well as possible [126]. The duration of inpatient neurological rehabilitation is typically 3 weeks and it should be initiated as early as possible. According to some authors, recovery of focal neurological deficits may occur within 24 months after surgery. Start of rehabilitation after this time interval is typically not indicated.

## 9 Results of current studies

### 9.1 Studies: anatomic simulations

#### 9.1.1 Orbital apex and middle cranial fossa

Bly et al. performed simulation of the transorbital approaches to different anatomical landmarks [27]. The simulation was based on a 3-dimensional CT dataset. This so-called virtual endoscopy was performed with iNtellect Cranial Navigation (version 1.1-14, Stryker Corporation, Kalamazoo, MI, USA). All 4 transorbital approaches according to Moe were tested, additionally the transnasal approach. The following criteria were applied:

1. General feasibility
2. Absence of neurovascular structures of vital importance in the corridor
3. Maximal angle between the manipulating instruments of 15°
4. Compared to the transnasal approach, the working angle relative to the skull base or to the sagittal plane deviates more than 15°
5. The working distance to the surgical target is significantly smaller than for the transnasal approach
6. In case access routes are combined, only transorbital approaches are used

Based on those criteria, the following surgical targets were analyzed: optic chiasm, cavernous sinus, trigeminal ganglion, superior orbital fissure, 3^rd^ ventricle, basal cistern, and clivus. Regarding the optic chiasm, the transnasal endoscopic approach fulfilled best the criteria, regarding all other targets, the transorbital approaches appeared more suitable. The authors then demonstrated the feasibility of these approaches to the listed targets in an anatomical specimen [27].

In another study, Bly et al. performed a computer simulation of approaching the lateral cavernous sinus, the apex orbitae, the trigeminal ganglion, and the base of the middle cranial fossa via the lateral retrocanthal incision. The insertion of the lateral canthal ligament could be preserved, the incision was made through the conjunctiva posterior of Whitnall’s tubercle. These authors also tested this simulated approach in anatomical specimens. The limitations of this study include that significant parameters could not be simulated, e.g. hemorrhage, soft tissue shift, differences between radiological and actual margins of pathology, or the dynamics of brain retraction. Up to now, a clinical study of this approach to access those central structures has not been published [27].

#### 9.1.2 Combined transnasal-transorbital approach

Ciporen et al. investigated the possibilities of the combined transnasal-transorbital approach in an anatomical model. The working angle and distance to important anatomical landmarks were examined: pituitary gland, optic chiasm, and cavernous segment of the ipsilateral carotid artery. The authors came to the conclusion that both the shorter working distance and the favorable working angle as well as the wider surgical field may represent an advantage of the transorbital approach in comparison to the transnasal approach, for example when accessing the following structures: pituitary gland, suprasellar region, clivus, and cavernous sinus. The authors emphasize that particular advantages result from a combination of the transnasal with the transorbital approach, e.g. with the 4-hands technique. The limitations of this study include the classical limitations of anatomical studies: the alterations of the soft tissues in the specimen, the deflated bulb, the shrinking of the cerebral parenchyma, absence of bleeding etc. Thus reliable clinical data have to be collected until the transorbital approaches to those far centrally located structures can be satisfactorily assessed [127].

### 9.2 Clinical trials

#### 9.2.1 Sinugenic complications

The management of sinugenic complications via transorbital approaches was analyzed by Lim et al. in a retrospective study of 13 patients. In 13/13 patients, the intervention was performed with neuronavigation.

In 7/13 patients, subperiostal or intraorbital abscesses of the orbital cavity were opened and drained. In 2/7 patients, simultaneous decompression of the optic nerve was performed for an orbital compression syndrome via the precaruncular approach. The optic nerve was decompressed from medially. 1/7 patients was treated for thrombosis of the cavernous sinus.

In 2/13 patients, an epidural abscess was drained through the upper eyelid approach. Craniectomy of the orbital roof was performed laterally. In both patients, the frontal sinus and the ethmoid were approached from the contralateral side via a transnasal endoscopic approach. 5/13 patients underwent treatment of a muco(-pyo-)cele, in 3/13 cases, the interfrontal septum was removed through the transorbital access. In all 13/13 patients, a complete regression of the clinical symptoms was described. CT imaging revealed findings that correlated with the clinical improvement. Limitations of this study include that exact data on the pre- and postoperative vision are missing. Measurements of the intraorbital pressure are not reported, indications and outcome of the orbital decompression cannot be exactly assessed. The study shows that patients with sinugenic complications can be treated successfully in a minimally invasive manner via transorbital approaches without important risks. Conclusions regarding exact indications, advantages and disadvantages compared to endonasal and transcranial approaches cannot be drawn from this study [39].

#### 9.2.2 Tumors of the frontal sinus

Kopelovich et al. describe the resection of an inverted papilloma and of two mucoceles of the frontal sinus via an upper eyelid incision. The frontal infundibulum was simultaneously opened endonasally in the sense of Draf II surgery. Complete resection of the pathology was achieved in 3/3 cases, the functional and cosmetic outcomes were described as excellent in 3/3 cases [128].

**Figure 15:**
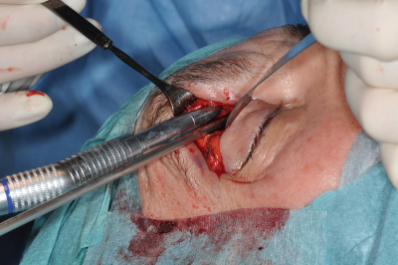
Endoscopically controlled access to the interfrontal septum. A segment of the anterior wall and the floor of the frontal sinus was temporarily removed.

The team around Moe et al. described the treatment of pathologies of the frontal sinus via transorbital approaches without relevant morbidity [39]. Regarding the access to the frontal sinus, the authors of the present discussion see significant advantages of the transorbital approaches. Depending on the individual anatomy, the access to the frontal sinus in transnasal endoscopic procedures remains limited to the medial and central aspects of the frontal sinus. Timperley et al. evaluated the lateral extent of the transnasal approach in an anatomical investigation. The authors first performed a Draf type III dissection and then they measured the lateral reach of the transnasal procedure. They could show important limitations of the transnasal approach to the lateral segments of the frontal sinus, especially the floor and the roof [129]. Hence, processes located in the lateral frontal sinus are frequently exposed via transfacial or coronal approaches, which require more collateral soft tissue dissection compared to transorbital approaches. Figure 15 (Fig. 15) depicts the access to the posterior wall of the frontal sinus after temporary removal of a segment of the anterior wall of the frontal sinus.

#### 9.2.3 Treatment of dura defects

The treatment of dural lesions was described by Moe et al. in 2 retrospective studies [1], [40]. Regarding the approaches, the authors distinguish between lesions of the interorbital and the supraorbital segment. The authors prefer the precaruncular incision to access the interorbital segment, and the upper eyelid incision to access the supraorbital segment. These authors recommend the transorbital approach for revision of transnasal procedures with persisting CSF leak. In cases of defects of the interorbital corridor, the authors use acellular dermis (Alloderm) or autologous fascia in a double layered fashion fixed with fibrin glue and BioGlue^®^ (CryoLife, Inc., Kennesaw, GA). In cases of defects of the supraorbital corridor, the authors perform a single layered repair.

In the first retrospective study (2009) [1], 12 patients were included. 6/12 had been referred to the department for revision after transcranial treatment (4 patients with 1 craniotomy, 2 patients with 2 craniotomies, 1 patient with 4 craniotomies). The first transorbital revision was successful in 12/12 cases [130].

In a second study (2011) [40], the authors report a patient population of 10 patients. In 8/10 patients, unilateral treatment was performed, in 2/10 bilateral surgery was performed. In 9/10 patients, the CSF leak stopped successfully, in 1/10 patients a recurrence occurred, which ceased with conservative therapy and did not require revision surgery. In a case report Raza and Boahene describe the reconstruction of a defect of the posterior wall of the frontal sinus and adjacent dura via an upper eyelid approach with temporary removal of a segment of the anterior wall of the frontal sinus. These authors mobilize the supraorbital nerve by opening the supraorbital foramen in inferior direction. The dura defect was closed with DuraGen^®^ (Integra Life Sciences, Plainsborough, NJ), a free fascia lata transplantation, and fibrin glue. The patient did not show any clinical hint to persisting rhinoliquorrhea 1 year after the intervention. It must be stated that confirmation by beta transferrin studies are not reported in those studies. The dural defects are heterogeneous regarding etiology, size, and location. A recommendation of an algorithmic procedure cannot be given. The conclusion that the transorbital approach is superior to a transcranial intervention seems to be justified with regard to the good results and the low morbidity even with the relatively low numbers of cases in those studies [130].

#### 9.2.4 Tumors of the anterior skull base

In a retrospective study, Andaluz et al. reported the resection of 3 tumors of the anterior skull base (3 female patients, medium age of 33 years, 2 suprasellar pituitary adenomas, and 1 craniopharyngioma). Abdel Aziz reported the resection of 9 tumors (7 meningiomas, 1 glioma, and 1 cavernoma) accessed via an upper eyelid approach after resection of a big superolateral bone segment [24]. The bone segment was repositioned by plate osteosynthesis. According to Andaluz et al. the average hospitalization amounted to 3 days, no complications were observed and the cosmetic result was excellent [28].

#### 9.2.5 Aneurysms of the anterior circulation

**Figure 16:**
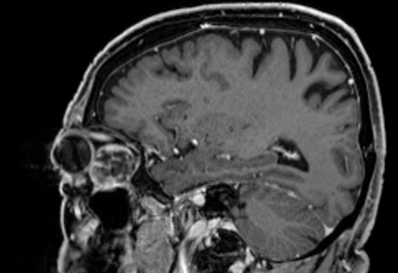
Retroorbital tumor of neuro-ectodermal origin. This 66-year-old patient presented with progredient diplopia and exophthalmos.

**Figure 17:**
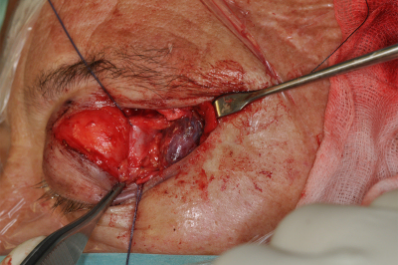
Exposure of the retrobulbar tumor via an upper eyelid approach. The resection was complete, a few days after intervention, exophthalmos and diplopia resolved completely.

In the study cited in 9.2.4, Andaluz et al. report the treatment 5 non-ruptured aneurysms of the anterior circulation after resection of a large superolateral bone segment via an upper eyelid approach. The average diameter of the aneurysm was 5 mm. The median duration of hospitalization was 2.2 days. Complications were not observed. The bone segment was repositioned by means of plate osteosynthesis. The cosmetic outcome was excellent 3 months after surgery [28]. Abdel Aziz et al. reported in a study of 40 patients about 31/40 patients who underwent treatment for aneurysm of the anterior circulation [24]. Abdel Aziz observed 4 complications, 1 hematoma of the eyelid, 2 infections, and 1 CSF leak. All complications were reversible. As discussed above, the studies of Andaluz and especially of Abdel Aziz do not meet the inclusion criteria of the present analysis, because the incision is drawn laterally beyond the bony orbital rim. However, to complement the topic, they are mentioned here as they excellently illustrate the enormous potential of the transorbital accesses. The authors of the present paper have released the lateral canthal ligament through a lateral extension of the supra-supratarsal incision for access to the lateral orbital wall and anterolateral skull base. By this access and after temporary resection of a bone segment, the retroorbital lesion (Figure 16 (Fig. 16)) of a 66-year-old patient with progressive exophthalmos and diplopia was completely extirpated (Figure 17 (Fig. 17)). There was no need for ICU admission, he was ambulatory the day of surgery, and diplopia and exophthalmos were completely resolved.

#### 9.2.6 Treatment of fractures of the anterior skull base

In a retrospective study, Moe et al. reported 8 patients treated anterior skull base fratures via the transorbital access. In the majority of cases, the indications for surgery were symptomatic fractures of the orbital roof resulting in diplopia or dystopia. In 7/8 patients, the orbital pathological findings resolved completely. The authors of the present have made excellent experiences with reconstruction complex fractures of the orbits and frontal sinuses via the transorbital approaches. Lateral extension and transorbital release of the canthal ligament allows for broad access. Transorbital endoluminal reposition of frontal sinus fractures is almost invariably feasible. The use of plates and screws can often be minimized, the use of mesh avoided. Endoscopic reposition of the fracture may in many cases be stabilized with application of glue or bone cement to the fractures fragments and postoperative splinting with a thermoadaptive cast over the frontal bone. In the authors’ experience, the transorbital approaches can replace the bicoronal access in the vast majority of cases of fracture management [1].

## 10 Discussion

Over the last 2 decades, surgery of the anterior skull base experienced an enormous development. Especially the introduction and enhancement of the transnasal endoscopic techniques promoted minimally invasive procedures. An astonishing spectrum of structures can now be reached. Snyderman et al. mention as borders of the coronal surgical corridor the orbital roof, the base of the middle cranial fossa, and the foramen jugulare. In the sagittal level, the corridor extends from the frontal sinus to the second cerebral vertebral body [131].

However, some important structures remain difficult to reach via the transnasal endoscopic approach. Those include important parts of the frontal sinus. The repositioning of fractures of the anterior and posterior wall of the frontal sinus is part of the routine spectrum of head and neck surgery. Often, those fractures are treated via a coronal incision [132]. This type of incision is a reliable approach with acceptable morbidity. It can be trained easily and experienced surgeons are able to complete the approach in short operative time. The most important risks are the damage of the frontal branch of the facial nerve and the development of alopecia, furthermore the loss of sensation. Many authors insert active drainage; often the approach requires hospitalization [133]. Additionally, numerous incisions are described that allow a direct transfacial access to the anterior wall of the frontal sinus, for example transpalpebral incisions, the modified Lynch incision, or the incision according to Siebenmann. Those approaches are performed in the skin of the forehead or the nose.

Those anatomical units are characterized by a skin type that is very likely to develop visible scars because of its texture. Incisions along or within the eyebrows are unfavorable because conspicuous scars occur due to the loss of hair. The incision within the skin of the eyelid represents the fundamental difference between the transfacial and the transorbital approaches. If the incision remains within the upper eyelid, the resulting scar is usually invisible. The extension in lateral direction into a periorbital fold remains invisible as well if this incision is not made beyond the orbital rim. The further extension of about 1.5 cm leads to a (generally less) visible scars lateral to the orbital rim. Such an incision was applied in the studies of Andaluz et al. and Abdel Aziz et al. discussed above [24], [28]. Those studies did not meet the inclusion criteria for transorbital approaches in two aspects: First, the incision reached into the facial skin with consecutively visible scarring, second the craniotomy was not only performed through the thin orbital roof, but through the thick bone of the calvarium. These studies were mentioned nonetheless, because they illustrate the enormous potential of the transorbital approaches. In those studies, a high number of non-ruptured aneurysm of the anterior circulation and tumors of the skull base were treated with particularly low morbidity.

The incision of the upper eyelid approach corresponds to the supratarsal incision utilized for cosmetic blepharoplasty. The authors of the present article varied the incision so that it follows the superior incision utilized in cosmetic blepharoplasty. This supra-supratarsal incision has two main advantages. First it extends the access in superior and lateral direction, second the supratarsal fold is preserved in its natural configuration. In this way, little inaccuracies remain nearly invisible which facilitates teaching of the method.

Without any question, the upper eyelid approach bears important risks. Structures that have to be exposed and protected frequently or regularly include the supraorbital bundle of vessels and nerves, the lateral canthal ligament, and the trochlea. Before temporarily removing a segment of the anterior wall and floor of the frontal sinus, the supraorbital nerve has to be mobilized. A removal of 2 segments that are split along the course of the nerve allows the effective protection of this nerve. The procedure and risk profile regarding the management of the supraorbital nerve should correspond with those of the coronal and transfacial approaches. Reliable data allowing a comparison of those approaches with regard to the morbidity of the supraorbital nerve could not be identified. 

The detachment of the lateral canthal ligament enormously extends the surgical corridor in lateral direction. Moe et al. favor the transconjunctival incision for accessing the lateral quadrant [26]. These authors preserve the origin of the lateral canthal ligament at Whitnall’s tubercle. The authors of the present article, however, favor detaching the lateral canthal ligament from endo-orbital. Thus it is possible to temporarily remove bigger bone segments and to achieve an access to the whole lateral anterior skull base, as similarly described by Andaluz and Abdel Aziz in their studies [24], [28]. Osteosynthesis plates should be adapted before completing the osteotomies in order to achieve an exact fitting of the repositioned segments. This approach allows an at least equivalent exposition in comparison to pterional craniotomy. The reconstruction of the lateral canthal ligament is performed as described by Moe [26]. The canthal ligament is looped via 2 drill holes in the orbital rim at the level of the zygomatico-frontal suture. When this reconstruction is precisely performed, the lateral canthus can be reconstructed without visible deformity. The risks of this measure encompass rounding of the lid angle, narrowing of the lid fissure, ectropion, entropion, epiphora, and kerato-conjunctivitis. 

The management of the trochlea is of paramount importance for the approaches to the interorbital corridor and the posterior aspects of the anterior skull base. Haug et al. show that the subperiostal release of the trochlea can be performed with low risk [25]. The team around Boahene observed a protracted but self-limiting postoperative diplopia in one patient. These authors had removed the trochlea via a precaruncular and transconjunctival access from medially. Observations of the authors of the present article in anatomical specimens and intraoperatively suggest that the subperiostal removal from lateral to medial is more reliable than from medial to lateral. This is a subjective observation that could not be confirmed by data. An anatomical study is currently under way. Raza et al. did not remove the trochlea in consecutive patients [2]. It will have to be clarified if the removal from medial vs. lateral makes a difference regarding the risk of diplopia. This could lead to preference of the upper eyelid over the precaruncular approach. The authors of the present article favor the upper eyelid approach with removal from lateral to medial. They consider it very important to release the trochlea because this measure widely opens the access to the anterior skull base and many advantages of the transorbital approach become apparent with this maneuver. Regarding reconstruction of the trochlea after atraumatic removal, most reports are consistent. In all relevant articles, fixation is not performed, the trochlea is repositioned with the orbital soft tissues. 

The low-risk management of the supraorbital nerve, the lateral canthal ligament, and the trochlea is key precondition for performing the transorbital approaches safely. A sound training of the operating and supervising surgeon with regard to oculoplastic, microsurgical techniques is very important before applying this method. This fact is also reflected in the literature on the value of interdisciplinary cooperation in skull base surgery. Furthermore, specialized training programs as they are offered for example by the European Academy of Facial Plastic Surgery (EAFPS.org) can contribute to an optimal outcome of this kind of surgery.

One Important limitation of the transnasal endoscopic procedure concern the technique of duraplasty. One factor correlating with successful management include the suprabasal positioning of the sealing material. The reasons are of biomechanical nature: the sealing material is compressed by the cerebral parenchyma in the defect and closes it in this way. This leads to an important theoretical advantage of transorbital techniques. Because of their obtuse working angle in relation to the skull base and the ability to perform craniectomy of large segments of the anterior skull base, broad access for the suprabasal insertion of sealing material can be created. After clipping the ethmoid arteries and resection of the fovea ethmoidalis, for example the olfactory fibers can be transected individually and the cribriform dura can be elevated up to and beyond the midline. Even an intradural procedure above the olfactory bulb was reported as well as simultaneous access from the contralateral side. This way it becomes possible to position sealing material across the entire cribriform plate. Also areas that are difficult to access by transnasal endoscopic technique can be reached, e.g. the entire posterior wall of the frontal sinus. Current data do not allow drawing a reliable conclusion if the transorbital approaches may actually improve the outcome of duraplasties. The major proportion of dural defects of the anterior skull base can be treated with excellent outcom through transnasal techniques. For example Moe et al. consider the transorbital approaches mainly for revision surgery in cases of persisting CSF leak after adequately performed transnasal repair or as an alternative to open craniotomy [40]. As an alternative to open craniotomy, the transorbital procedure has important theoretical advantages: the access is more direct, the manipulation of bone is less extensive, retraction of the brain is avoided, and often the olfactory fibers can be protected more selectively. If those theoretical advantages really improve the outcome in comparison to open procedures cannot be confirmed based on the currently available data. The best available evidence, however, allows the conclusion that a transorbital procedure should be considered if it represents a feasible alternative to transcranial repair.

The management of tumors of the anterior skull base follows similar reflections as that of dural leaks. The transorbital approach is appropriate when the transnasal access is limited and morbidity of the transcranial transcranial approach should be avoided.

The analysis of the transorbital approaches experiences important limitations both in the available body of literature as well as in the present review. Available studies are nearly exclusively retrospective and summarize the experiences of single surgeons. The quality of the surgeons is an important parameter that is not included in the evaluation of the data. The number of cases published until now is rather small. Evaluations of how this method may be trained and how the learning curve develops, do not exist.

Methodical weaknesses of some studies are apparent. Computer simulations and anatomical studies can show the general feasibility of approaches and techniques. Their application and comparison with established techniques has not been studied in the clinical setting in many instances. The retrospective conclusion regarding the treatment of CSF leaks are based on clinical observation. Results of laboratory analysis, e.g. beta transferrin are not presented. The results of decompression of the orbita and the optic nerve are not precisely distinguished, even though these 2 represent fundamentally different entities. Studies with long-term follow-up regarding the possible incidence of frontal sinus outflow obstruction are currently not available. Regarding the relevance of diagnostic, technical, and staff-related preconditions, peri- and postoperative care, there are no specific data present. Therefore, the experiences with open craniotomy and transnasal techniques are extrapolated and interpreted. 

Many of the above-mentioned limitations, however, also apply to the literature of established procedures. The majority of articles on e.g. transnasal endoscopic skull base surgery is retrospective, summarizes the experiences of single surgeons, and allows only rarely a reliable comparison to other methods.

New surgical methods develop principally on the basis of published experiences of only few authors. They gain attention and are implemented more and more if the advantages are understandable, feasible, and finally have a clear advantage for the physician and patient.

The technique of transorbital skull base surgery has an enormous potential. In many cases, it allows accessing pathologies with decreased collateral soft tissue and minimal bone dissection compared to open techniques. The patients are often ambulatory the day of surgery, surveillance in intensive care units is rarely required. Compared to the transnasal approach, few but important advantages are apparent, especially in the context of reaching important anatomical structures.

The best available evidence justifies the well reflected indication of transorbital approaches, especially as alternative to the transcranial procedure. The interdisciplinary cooperation in large centers and the ability to change to an open transcranial procedure should be a precondition for performing these approaches.

## 11 Conclusion

The transorbital approaches to the skull base complete the armamentarium of surgical techniques of the anterior skull base. Available data up to now indicate that this method is characterized by a low risk profile and by important advantages when accessing several pathologies compared to established methods. Those advantages include the protection of the endonasal system of paranasal sinuses and their physiological functions, the relatively limited soft tissue dissection, and the relatively short distance to reaching the pathology. An interdisciplinary approach contributes to optimal care of the patient and to achieving the best possible results. The best available evidence justified the well-reflected indication of transorbital approaches especially as alterative to the transcranial procedure.

## Notes

### Competing interests

The authors declare that they have no competing interests.
